# Supplementary material for: A UMLS-based spell checker for natural language processing in vaccine safety
Source: BMC Med Inform Decis Mak. 2007 Feb 12;7:3. doi: 10.1186/1472-6947-7-3 (PMC1805499; doi:10.1186/1472-6947-7-3)
Supplement: Additional file 13 — RAP application directory. Sets up directory for RAP (RDF application for PHP) source code [file 1472-6947-7-3-S13.gz › rap/doc/phpdoc/default/RDF_RES.html]

xml version="1.0" encoding="iso-8859-1"?


Docs For Class RDF\_RES


## Class RDF\_RES

Description

Description |
Methods (details)

Located in /vocabulary/RDF\_RES.php (line 20)

Method Summary

Description |
Methods (details)

void
ABOUT
()

void
ABOUT\_EACH
()

void
ABOUT\_EACH\_PREFIX
()

void
ALT
()

void
BAG
()

void
BAG\_ID
()

void
DATATYPE
()

void
DESCRIPTION
()

void
FIRST
()

void
ID
()

void
LI
()

void
LITERAL
()

void
NIL
()

void
NODE\_ID
()

void
OBJECT
()

void
PARSE\_TYPE
()

void
PARSE\_TYPE\_RESOURCE
()

void
PREDICATE
()

void
PROPERTY
()

void
RDF\_LIST
()

void
RESOURCE
()

void
REST
()

void
SEE\_ALSO
()

void
SEQ
()

void
STATEMENT
()

void
SUBJECT
()

void
TYPE
()

Methods

Description |
Methods (details)

ABOUT (line 113)

void

ABOUT
()

ABOUT\_EACH (line 119)

void

ABOUT\_EACH
()

ABOUT\_EACH\_PREFIX (line 125)

void

ABOUT\_EACH\_PREFIX
()

ALT (line 23)

void

ALT
()

BAG (line 29)

void

BAG
()

BAG\_ID (line 131)

void

BAG\_ID
()

DATATYPE (line 173)

void

DATATYPE
()

DESCRIPTION (line 101)

void

DESCRIPTION
()

FIRST (line 77)

void

FIRST
()

ID (line 107)

void

ID
()

LI (line 161)

void

LI
()

LITERAL (line 149)

void

LITERAL
()

NIL (line 59)

void

NIL
()

NODE\_ID (line 167)

void

NODE\_ID
()

OBJECT (line 95)

void

OBJECT
()

PARSE\_TYPE (line 143)

void

PARSE\_TYPE
()

PARSE\_TYPE\_RESOURCE (line 155)

void

PARSE\_TYPE\_RESOURCE
()

PREDICATE (line 89)

void

PREDICATE
()

PROPERTY (line 35)

void

PROPERTY
()

RDF\_LIST (line 53)

void

RDF\_LIST
()

RESOURCE (line 137)

void

RESOURCE
()

REST (line 71)

void

REST
()

SEE\_ALSO (line 179)

void

SEE\_ALSO
()

SEQ (line 41)

void

SEQ
()

STATEMENT (line 47)

void

STATEMENT
()

SUBJECT (line 83)

void

SUBJECT
()

TYPE (line 65)

void

TYPE
()

Documentation generated on Fri, 17 Dec 2004 16:17:23 +0100 by phpDocumentor 1.3.0RC3
